# A new class of optical sensors: a random laser based device

**DOI:** 10.1038/srep35225

**Published:** 2016-10-11

**Authors:** Emilio Ignesti, Federico Tommasi, Lorenzo Fini, Fabrizio Martelli, Niccolò Azzali, Stefano Cavalieri

**Affiliations:** 1 Dipartimento di Fisica e Astronomia, Università di Firenze, Via G. Sansone 1 I-50019 Sesto Fiorentino, Italy

## Abstract

In a random laser the optical feedback is provided by scattering rather than by an optical cavity. Then, since its emission characteristics are very susceptible to the scattering details, it is a natural candidate for making active sensors to use as a diagnostic tool for disordered media like biological samples. However, the methods reported up to now, requiring the injection of toxic substances in the sample, have the drawback of altering the physical-chemical composition of the medium and are not suitable for *in-vivo* measurements. Here we present a random laser based sensor that overcomes these problems by keeping gain and diffusion separated. We provide an experimental characterisation of the sensor by using a reference diffusive liquid phantom and we show that, compared to a passive method, this sensor takes advantage of the gain and spectral properties of the random laser principle.

In the last two decades the development of optical sensors for the study of diffusive materials has made great advances and in particular the interest for the investigation of biological samples has generated a wealth of different techniques and applications^1^,^2^,^3^. In addition, the scattering properties of the materials are not always considered as undesired loss effects and noise sources; indeed, engineered disorder was also introduced in photonic devices in order to increase sensitivity^4^. While all these different techniques are based on passive optical methods, some researchers have explored the potentialities of stimulated emission to enhance the detection sensitivity by coupling a gain material with an optical microcavity^5^,^6^. Measurements of transport mean free path by coupling a diffusive sample with an amplified spontaneous emission beam from a dye solution pumped by a Nd: YAG laser have also been reported^7^. An other kind of active device can be obtained by exploiting the physical principle of the random laser^8^. Since the pioneer work of Letokhov^9^ it has been recognised that a diffusive material, when coupled to a gain medium, can provide the necessary optical feedback to sustain a laser action. Respect to a conventional laser, this light source does not need an optical cavity: the stimulated emission mechanism, that acts along random paths of light inside the disordered medium, can lead to laser-like emission if gain overcomes losses. Since ‘80s different random laser systems have been proposed. Many of them are obtained by embedding in a liquid or solid matrix active centres like dye molecules or Ti:Sa powders, and scatterers like ZnO or TiO_2_ nanoparticles or powdered glass^10^,^11^,^12^.

Biological tissues form an important class of scattering materials, and this kind of optical media have been found suitable for generating random laser radiation. Random laser action has been reported in several *ex-vivo* biological structures infiltrated with amplifying dye molecules: chicken tissue and pig fat^13^, rat muscle^14^ and chicken breast tissue^15^, bovine bone^16^, wing of cicada covered with a layer of a dye doped polymer film^17^ and butterfly embedded with ZnO nanoparticles^18^. Moreover, using a drug-dye composite as active medium infiltrated within the tissue, the possibility to develop new combined opto-chemical therapies has been recently reported^19^.

Notably and from diagnostic point of view, in *ex-vivo* dye infiltrated human tissue, emission spectrum modifications have been reported in malignant tissue respect to the healthy one^20^, opening the interesting opportunity to exploit the random laser physics to design new sensors to detect biological tissue diseases. An *ex-vivo* dye infiltrated human tissue from the same individual has been used for mapping the cancerous and healthy zones^21^.

The striking point is that the typical emission features of a random laser strongly depend on the scattering properties of the medium^22^,^23^,^24^,^25^,^26^,^27^, as well as the geometry of the active zone^28^ and the pump profile^29^. Indeed this emission is extremely sensible to small scale alterations in dimension, distribution and optical response of the scatterers. Hence, the random laser principle has been recognised as a great opportunity to create a new kind of active optical sensors for different scattering media^30^,^31^. Since the stimulated emission mechanism alters the emission spectrum shape, the effect of the disorder can be detected by measurements of spectral properties variations, instead of by energy measurements or light path reconstruction as in passive systems. In addition, in a random laser the scattering does not play the role of a loss factor, as in many passive methods, but it actually feeds the signal, leading to more suitable strategies for sensing strong scattering samples. Then, respect to typical passive techniques, a random laser based sensor can potentially take advantage of amplification of weak signals and of a detection strategy with lower experimental and computational difficulties. However, up to now the necessity to inject toxic substances, like dye molecules and solvents in the disordered medium, has hindered the application of random laser sensors for a real competitive utilisation on biological samples, given the impossibility to perform *in-vivo* measurements.

In the following we describe a random laser based sensor that does not suffer of this limitation. Our idea to avoid the injection of external substances in the scattering sample is to *put the active medium out of the disorder*. Then, our design consists in a transparent spherical cell with an active medium, leaving the sensor to communicate with the external disordered medium through the emitted and received light (Fig. 1).

The transparent walls of the cell allow the propagation of the spontaneously emitted radiation to the external disordered medium. After a random path through the diffusive medium, an amount of light can go back to the cell, thus undergoing amplification by stimulated emission in the active medium. The external disordered medium provides a feedback mechanism for the spontaneously emitted radiation, in a way dependent on the characteristics of the single scatterers and on their concentration as long as the single scattering approximation holds. Moreover, the optical fibre that carries the pump beam to create the population inversion also collects the output signal. This gives to our device characteristics of high portability and manageability.

## Results

### Sample preparation

In the measurements reported in this paper the disordered scattering medium is an aqueous dilution of Intralipid^®^ 20%, a fat emulsion composed by suspended particles with size ranging from 50 to 700 nm^32^. Measurements were performed for several values of the pump energy and several values of the concentrations of scatterers obtained by different dilutions of Intralipid^®^ 20%. This substance is widely used to prepare reference tissue-like phantoms^33^,^34^,^35^.

In order to characterize the scattering properties of the different dilutions, the chosen key parameter is the light transport mean free paths *L*_*T*_, i.e. the average distance beyond which the propagation direction of the propagating photons becomes completely randomized. It differs from the scattering mean free path by a factor that depends on the asymmetry of the scattering phase function. In our prepared samples, the used concentrations correspond to different *L*_*T*_s that cover a very large range of lengths, from a few mm to 60 μm, measured at the reference wavelength of 632.8 nm.

The reference signal was collected from an external sample without Intralipid^®^ 20% (pure water) and it mainly consists in photons directly emitted from the active medium to the optical fibre with the additional contribute of the Fresnel reflections at the cell walls.

### Experimental spectra

Figure 2 shows typical spectra of the signal obtained at a fixed pump energy of 4.32 ± 0.04 mJ and for eight different dilutions of Intralipid^®^ 20% in the external medium. Compared to the reference signal measured in pure water, even the weakest scattering medium (*L*_*T*_ = 2.75 mm) has the effect of modifying the shape of the spectrum, that appears still broad but with a peak at longer wavelengths (at ~600 nm) respect to the fluorescence peak (at ~590 nm); increasing the scatterers concentration, this peak shifts toward the red wavelengths up to ~625 nm. The increase of concentration leads to two other main features: a large enhancement of the total emission and the narrowing of the spectrum itself. These effects are peculiar of the random laser process. Because of the stimulated emission, at high energy the overall FWHM of the spectra changes from 23 (pure water) to 10.5 nm (*L*_*T*_ = 0.06 mm).

In Fig. 3 the peak value of the output spectrum, as a function of the pump energy and for the different Intralipid^®^ 20% dilutions, is reported together with the reference signal (pure water). The signal shows an enhancement in presence of strong scattering with a detection limit of the method that is roughly represented by the sample with *L*_*T*_ of 2.75 mm. An even better accuracy is provided by the analysis of the spectral behaviour of the emission. The peak wavelengths as a function of pump energy are reported in Fig. 4 for the same *L*_*T*_ values of Fig. 3. The peak redshift is strongly dependent on the concentration and, notably, the redshift reaches a saturation value that solely depends on the scatterers concentrations in the external medium, whatever the intensity of the pump energy.

### Comparison with a passive case

In order to highlight the efficiency of the active random laser sensor, a comparison with a passive one with a similar structure was performed. To simulate a passive device, the sensor was removed from the optical fibre and the pump laser substituted by a diode laser with a broad emission spectrum (20 nm FWHM) centred at the wavelength of 650 nm. Hence, the optical fibre was immersed in the external medium and the collected scattered light was extracted and sent to the spectrometer by a beamsplitter. The measurements were obtained by collecting the cw signal out of the fibre for 17 ms. In this passive case only modifications of the spectrum intensity were observed, with no shift or spectral shape changes, confirming the fact that the shift of the peak is then peculiar of the active sensor.

The comparison between the behaviour of the two systems is shown in Fig. 5, where the measured spectral peak intensities are reported as a function of *L*_*T*_ in the external medium. Both curves are normalised to the corresponding reference signal obtained with pure water. The active device presents a much stronger increase of signal when *L*_*T*_ becomes shorter, up to more than one order of magnitude. The reason stands in the combined effect of total emission increase and spectral narrowing that is typical of the random laser emission.

The large modification in the spectral behaviour, due to the interplay between the external feedback from the disordered medium, the losses by re-absorption and the gain by stimulated emission, undoubtedly represents the main effect that distinguishes this random laser based sensor from alternative passive systems.

## Discussion

In conclusion we have demonstrated that the gain characteristics and spectral properties of the random laser can be usefully exploited in optical sensing applications. Maintaining the active part of the device separated from the diffusive one allowed us to build a self-contained optical sensor that could be employed in non-invasive diagnostic of biological samples. It is worth to emphasise that the results show an high sensitivity, (modifications of a few tens of μm in *L*_*T*_ are well resolved), together with the capability to sense the transport mean free path lengths, with the same sensor, for values ranging for almost two order of magnitude. A further peculiar feature of this active sensor is the redshift of the spectrum peak, which, at high pumping energy, is determined only by the scattering properties of the external medium.

Regarding future applications on tissue optics, it is important to stress that the pumping beam at 532 nm is completely absorbed by the active medium, whereas the light that actually propagates through the medium is a low intensity fluorescence that is safe even for *in-vivo* applications.

## Methods

### Experimental set-up

**Figure 1:**
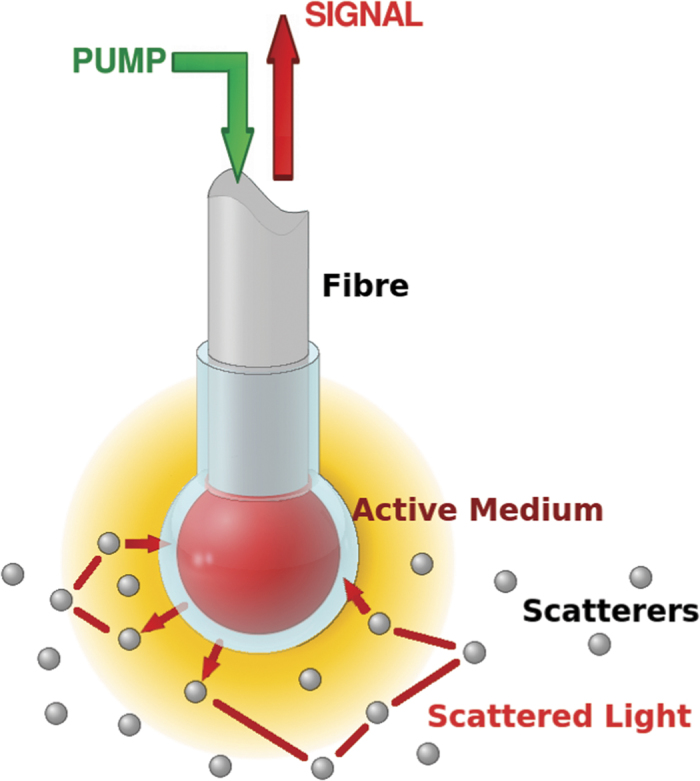
The optical fibre ends with a transparent spherical cell containing the active medium. The pump beam and the output signal are transported by the same fibre.

**Figure 2:**
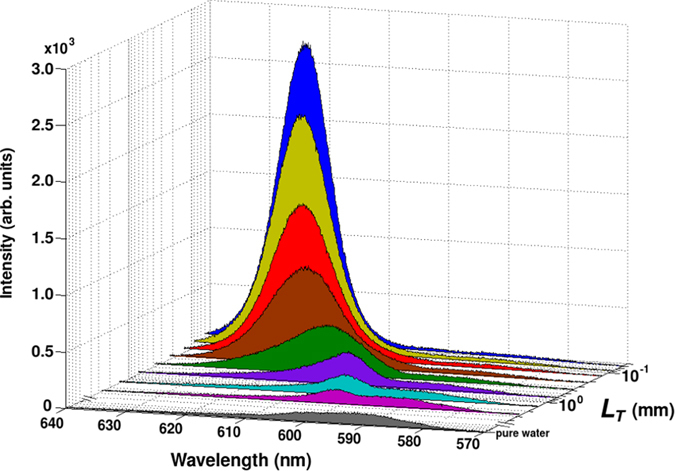
Typical spectra for different values of the transport mean free path *L*_*T*_ in the external disordered medium at a fixed pump energy of 4.32 ± 0.04 mJ. As the scatterers concentration increases, i.e. as *L*_*T*_ decreases, the peak presents both a monotone enhancement and a red-shift from 590 nm in pure water to 625 nm for *L*_*T*_ = 0.06 mm.

**Figure 3:**
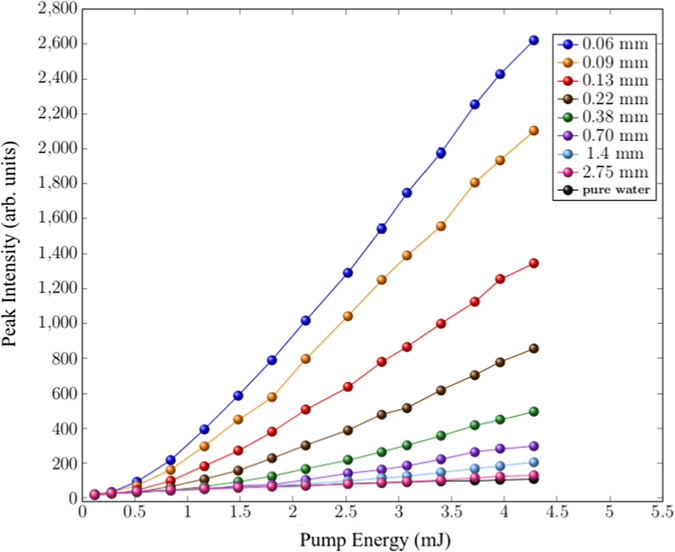
Intensity of the signal peak as a function of pump energy for different values of the mean free path *L*_*T*_ (values shown in legend). The reference signal at zero concentration of scatterers (pure water) is also reported.

**Figure 4:**
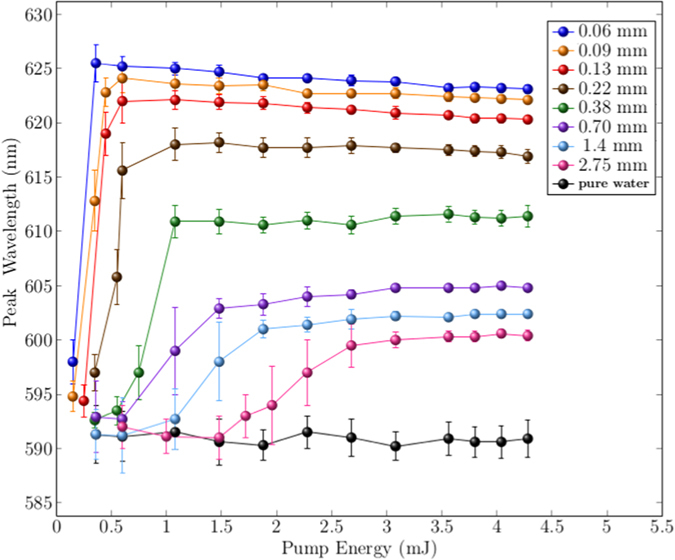
Shift of the spectrum peak as a function of pump energy for different scatterers concentrations. The peak shifts toward longer wavelengths reaching a saturation value, that only depends on *L*_*T*_ of the external medium. Notably, no red-shift is detected in the case without scatterers (pure water).

**Figure 5:**
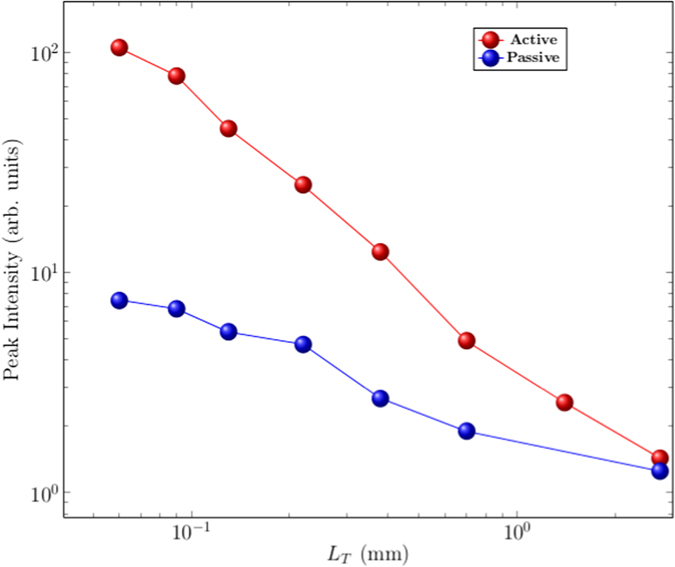
Comparison between the random laser active sensor and a passive one. The peak values of relative spectra are reported, normalised to the value measured with pure water. The passive system consists of a diode laser beam (with a broad emission spectrum of 20 nm) coupled with an optical fibre whose head is immersed in the external disordered medium. The pump energy for the active case is 4.32 ± 0.04 mJ.

**Figure 6:**
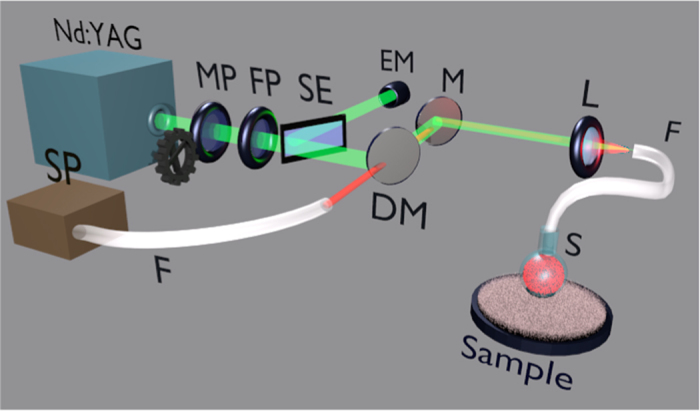
Scheme of the experimental set-up: Nd:YAG: pump beam source; SP: spectrometer; MP: movable polariser; FP: fixed polariser; SE: semi-transparent plate; EM: Energy Meter; DM: dichroic mirror; M: mirror; L: lens; F: optical fibre; S: sensor. The green beam is the pump and the red one the signal.

The structure of the sensor is schematically shown in Figs 1 and 6. The sensor head was obtained by a glass capillary with a refractive index@632.8 nm of 1.4709 ± 0.0002, measured by a semi-automatic instrument (COMPASSO)^36^,^37^,^38^,^39^ and based on a prism coupling technique and dark line spectroscopy.

The hollow sphere of an external diameter of few mm was realised in a closed end of the capillary by glass-blowing. Then the sphere was filled with the active medium and the optical fibre of a core of 900 μm was inserted in the opposite side. The active medium is composed by a suitably designed concentration of dye molecules and scatterers in an alcoholic solution. The whole device is covered by patent^40^.

In Fig. 6 a schematic diagram of the experimental set-up is shown. The pump beam, provided by a frequency doubled Q-switched Nd:YAG at a repetition rate of 2 Hz, is directed and focalised to the input of the sensor fibre, with a coupling efficiency of about 32%. The pumping energy is tuned by a pair of polarizers, the first of which is movable by a stepper motor connected to a PC, while the second one remain fixed. A reflection from a semi-transparent plate is sent to an energy meter to indirectly measure the pumping energy. The sensor head is immersed inside the sample, that is the external liquid medium, during the measurements.

The radiation emitted by the sensor is collected by the same fibre and sent back to a dichroic mirror towards a spectrometer with resolution of 0.25 nm. The shot-to-shot spectra are then automatically acquired and stored by a PC.

### Determination of the transport mean-free-path

The values of *L*_*T*_ for different dilutions of Intralipid ^®^ 20% have been obtained from the expression for the reduced scattering coefficient 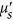 (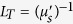) at the reference He-Ne laser wavelength 632.8 nm^41^,^42^:


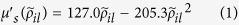


where 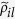 is the fractional volume occupied by the scatterers, given the different components of the emulsion^33^. 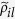 is related to the volume concentration of Intralipid^®^, *ρ*_*il*_, i.e. the ratio between the volume of Intralipid^®^ 20% and the total volume of the emulsion (Intralipid^®^ and water), by the relation 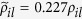
42. The equation also accounts for the deviation from the linearity between 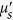 and *ρ*_*il*_ due to the lack of validity of the independent scattering approximation, that strictly holds only when the average inter-particles distance is much larger than the wavelength.

## Additional Information

**How to cite this article**: Ignesti, E. *et al*. A new class of optical sensors: a random laser based device. *Sci. Rep.* **6**, 35225; doi: 10.1038/srep35225 (2016).
